# First-Trimester Pregnancy Tumour of the Anterior Maxillary Gingiva: A Case Report of Lobular Capillary Hemangioma

**DOI:** 10.7759/cureus.116970

**Published:** 2026-09-26

**Authors:** Suresh Chinnakutti, Karthik Rajaram Mohan, Selvalakshmi Saravanan, Vijayapriyangha Senthilkumar, Harikaran Jeyakkodi

**Affiliations:** 1 Oral and Maxillofacial Surgery, Vinayaka Mission's Sankarachariyar Dental College, Vinayaka Mission's Research Foundation (Deemed to be University), Salem, IND; 2 Oral Medicine and Radiology, Vinayaka Mission's Sankarachariyar Dental College, Vinayaka Mission's Research Foundation (Deemed to be University), Salem, IND; 3 Periodontology, Vinayaka Mission's Sankarachariyar Dental College, Vinayaka Mission's Research Foundation (Deemed to be University), Salem, IND; 4 Conservative Dentistry and Endodontics, Vinayaka Mission's Sankarachariyar Dental College, Vinayaka Mission's Research Foundation (Deemed to be University), Salem, IND

**Keywords:** anterior maxilla, gingiva, pregnancy tumour, radiovisiography, . surgical excision

## Abstract

Pregnancy tumour, biologically known as lobular capillary hemangioma or pyogenic granuloma, is a benign vascular lesion that typically arises on the anterior maxillary gingiva during the second or third trimester of pregnancy. Driven by elevated levels of estrogen and progesterone alongside local chronic irritation, these lesions can cause significant discomfort, localised bleeding, and masticatory difficulty. We report the case of a 21-year-old female at the third week of gestation presenting with a rapidly enlarging, painless, pedunculated reddish mass or growth on the anterior maxillary gingiva. Management consisted of conservative surgical excision under local anaesthesia. Histopathology confirmed the clinical diagnosis of lobular capillary hemangioma. This case highlights the importance of distinguishing pregnancy tumours from malignant oral neoplasms, practising conservative management during pregnancy, and maintaining meticulous periodontal care.

## Introduction

A pregnancy tumour, also termed pregnancy-associated pyogenic granuloma, granuloma gravidarum, or epulis gravidarum, is a benign, reactive vascular lesion of the oral mucosa rather than a true neoplasm [1,2]. It occurs in up to 5% of pregnancies, most often during the second or third trimester when circulating estrogen and progesterone are maximal, and arises predominantly from the interdental papilla of the anterior maxillary gingiva as a solitary, pedunculated or sessile, smooth-to-lobulated mass [2]. Its pathogenesis is multifactorial: gestational hormonal surges amplify angiogenic and inflammatory signalling in gingival tissue already irritated by plaque, calculus, or minor trauma, producing the lesion’s characteristic rapid growth, prominent vascularity, and tendency to bleed with minimal provocation [2,3]. 

Pregnancy-associated pyogenic granuloma most commonly manifests during the second or third trimester, coinciding with peak circulating estrogen and progesterone levels believed to potentiate angiogenesis and an exaggerated inflammatory response to local gingival irritants [1,2]. First-trimester presentation, as in the present case, is comparatively uncommon and poses a distinct diagnostic dilemma: clinicians may be less inclined to attribute an early gestational gingival growth to hormonal pregnancy-associated change, increasing the risk that the lesion is initially misclassified as a traumatic fibroma, peripheral ossifying fibroma, or peripheral giant cell granuloma before histopathological confirmation [1,2]. This early onset also raises the clinical prospect of a longer period of hormonal exposure before parturition, during which the lesion may continue to enlarge, alongside heightened caution regarding any proposed intervention given the greater physiological sensitivity of the first trimester relative to later pregnancy [1,2].

The conventional management approach for pregnancy pyogenic granuloma is conservative, favouring watchful waiting with reinforcement of oral hygiene and deferral of definitive surgical treatment until after parturition, since many lesions partially or completely regress once hormone levels normalise postpartum [2]. Surgical intervention during pregnancy is generally reserved for lesions causing significant functional interference, recurrent bleeding, or substantial patient distress. In the present case, surgical excision was undertaken despite the ongoing pregnancy because the lesion was causing considerable patient discomfort and a marked aesthetic concern in the anterior esthetic zone, factors judged to outweigh the risks of proceeding with intervention during the first trimester rather than continuing conservative management alone. The complications of untreated pyogenic granuloma include recurrent bleeding, ulceration and secondary infection, functional interference with mastication and oral hygiene, and esthetic disfigurement [3].

We report a case of pregnancy tumour of the anterior maxillary gingiva in a primigravida during the first trimester, emphasising its clinical diagnosis, differentiation from other reactive and neoplastic gingival lesions, and conservative surgical management.

## Case presentation

A 21-year-old female, gravida 1 para 0 (G1P0), currently in the first trimester of pregnancy, presented with a chief complaint of a growth in the gums in the upper front teeth region. This was her first occurrence of such a growth. She reported that it began as a small growth approximately 2 months prior to presentation and had gradually progressed to its present size. The growth was painless and did not interfere with mastication or speech; however, the patient was concerned about its unaesthetic appearance, which prompted her to seek treatment. No triggering event, such as trauma or recent dental treatment, was recalled at the onset. There was no history of consanguineous marriage, and no family members had experienced a similar gingival growth. General examination revealed her vital signs, including blood pressure, pulse, and temperature, to be within normal limits. Medical history revealed she was in the first trimester of pregnancy. She had no comorbidities like diabetes, hypertension, or thyroid disorder. She had no history of any drug allergy. She was on vitamin, iron, and folic acid supplements for her first trimester of pregnancy. She had no history of tobacco chewing, betel nut chewing, or smoking. On intraoral clinical examination, on inspection, a growth was present involving the labial aspect of the marginal and attached gingiva, measuring approximately 1.5 × 1 cm. The lesion bled on provocation; however, there was no generalised bleeding on probing elsewhere in the oral cavity. The anterior and inferior parts of the growth had reddish areas, and the surface appeared shiny (Figure 1).

**Figure 1:**
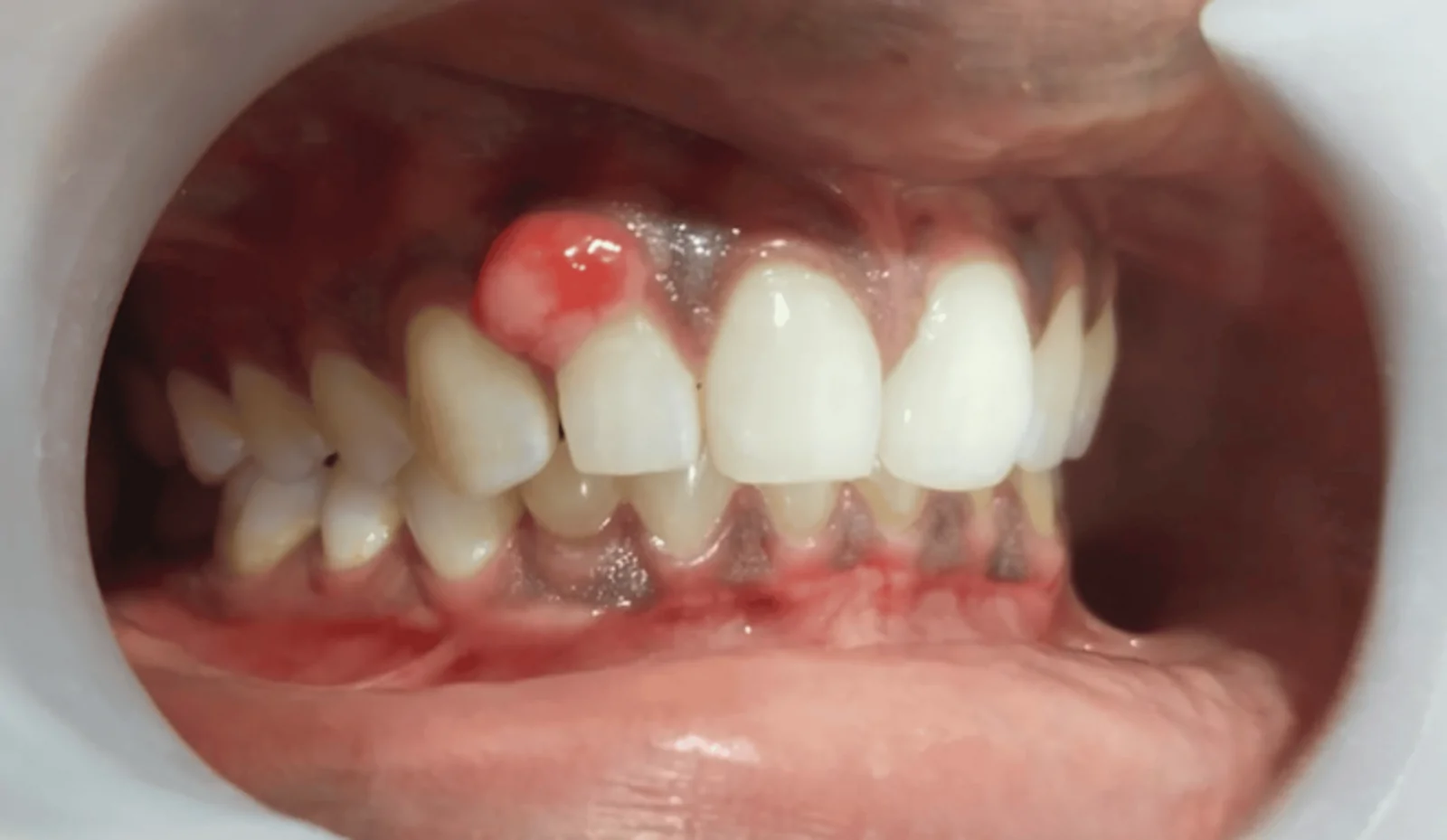
Intraoral clinical examination revealed growth involving marginal and attached gingiva in relation to right maxillary lateral incisor and canine tooth The anterior superior and inferior surfaces of the gingival growth had reddish areas with a shiny surface.

The tooth in the vicinity of the growth was free of supragingival calculus. On palpation, it was soft in consistency and showed a positive modified diascopy test with gentle pressure from the back end of the mouth mirror (blanching). A radiovisiography taken in relation to the growth revealed the absence of any subgingival calculus deposits on the teeth in the vicinity of the gingival growth (Figure 2).

**Figure 2:**
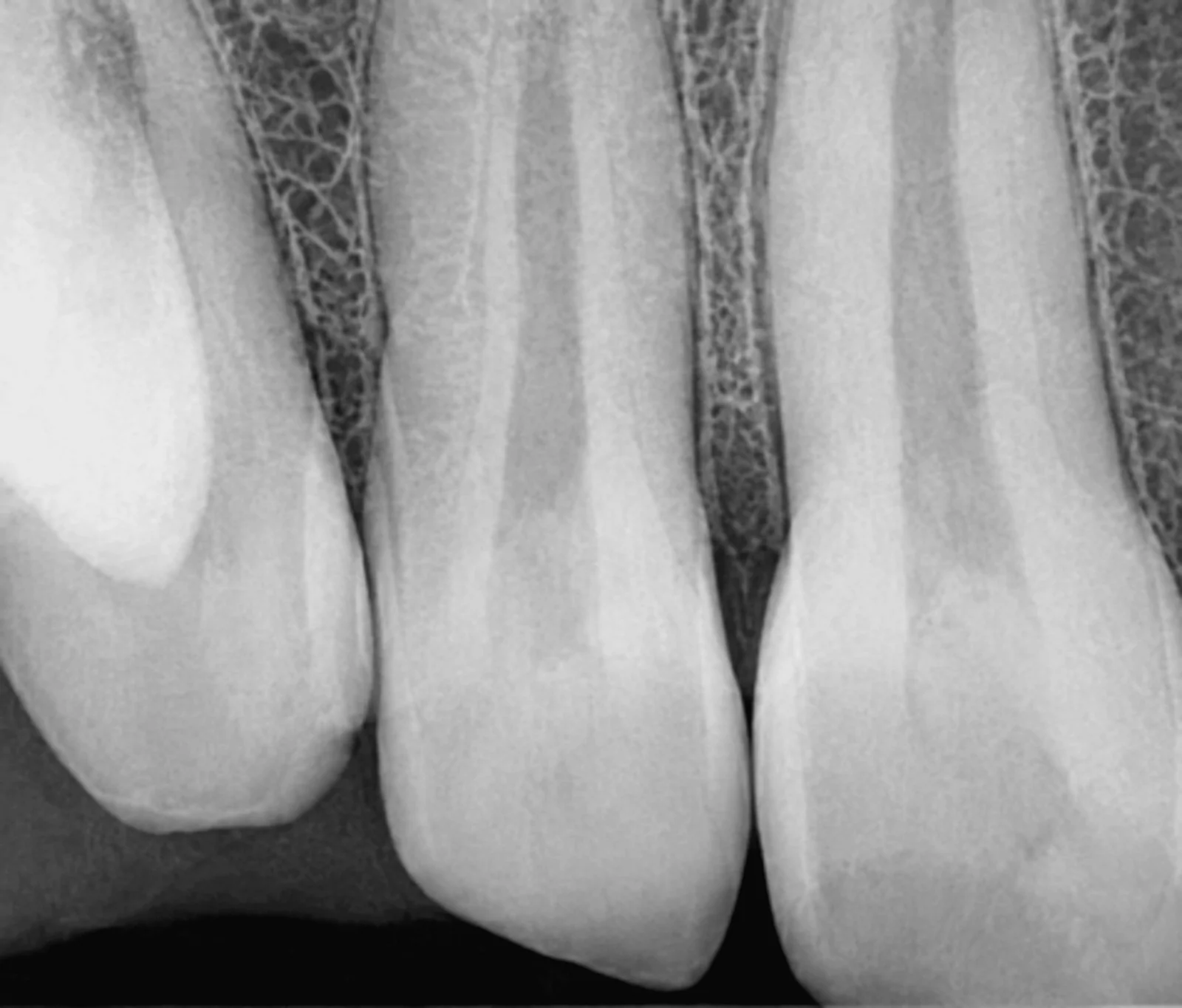
Radiovisiography (RVG) in relation to right maxillary lateral incisor revealed no calculus deposits near the cementoenamel junction on the tooth

Based on the history and the above clinical, radiographic and laboratory findings, a provisional diagnosis of pregnancy tumour involving the gingiva was made. The differential diagnosis includes pyogenic granuloma, peripheral ossifying fibroma, and peripheral giant cell granuloma. Pyogenic granuloma bleeds profusely even with slight provocation. The absence of bleeding on slight provocation and supra- or subgingival calculus on the teeth excluded pyogenic granuloma. On radiographs, peripheral ossifying fibromas have focal areas of calcification within the growth, which were absent in our case. Hence, peripheral ossifying fibroma was ruled out. Peripheral giant cell granulomas, though they appear on the gingiva, have a slight bluish tinge and usually cross the midline. The lack of a bluish tinge and the absence of infection-related pain ruled out peripheral giant cell granuloma. 

The laboratory diagnosis of a high oestrogen level of 692 pg/mL (normal range 150-300 pg/mL) by the electrochemical immunoassay (ECLIA) method confirmed the clinical diagnosis. The growth was excised under infiltration with 2% lignocaine local anaesthesia (Figure 3).

**Figure 3:**
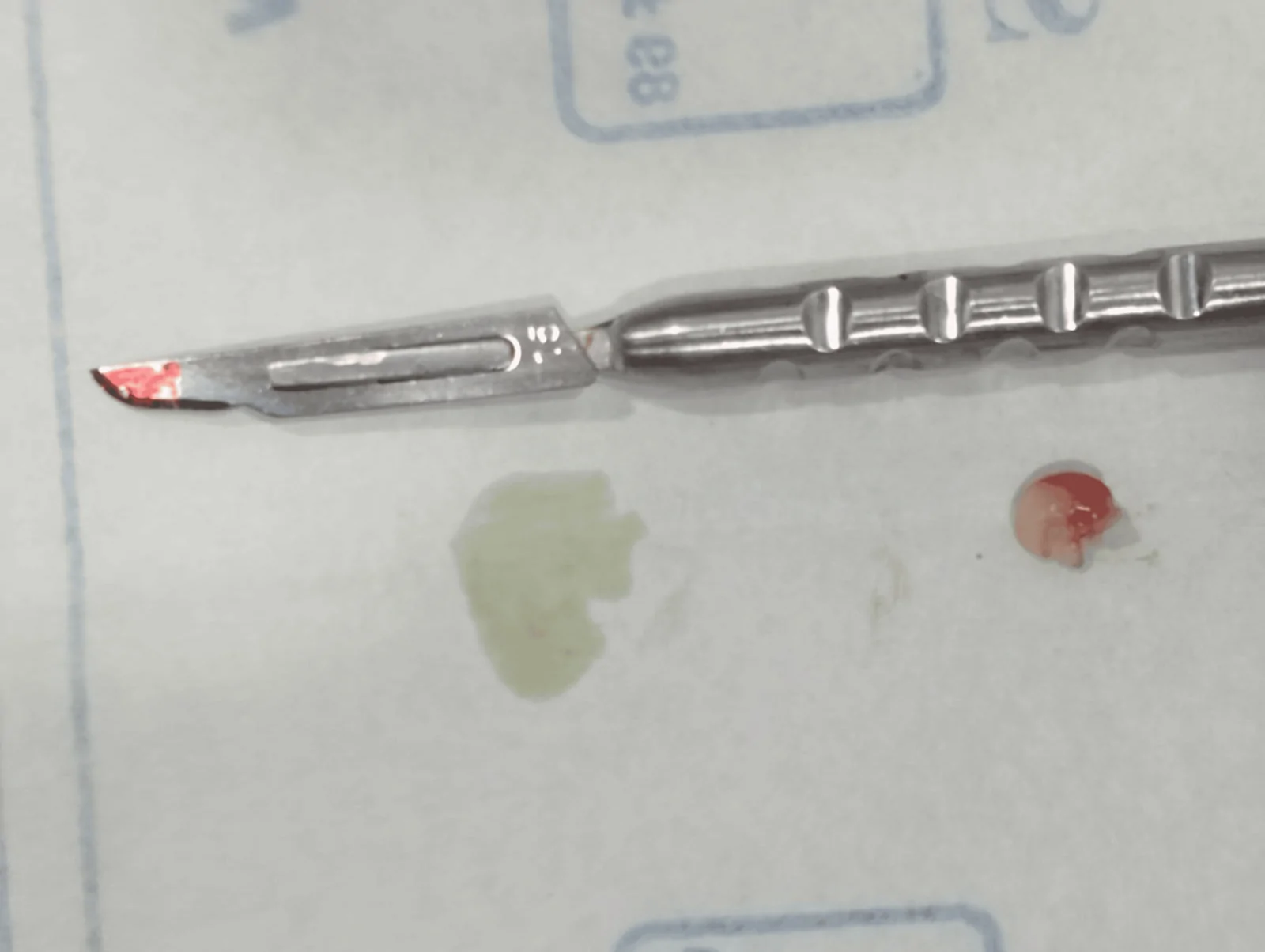
Excised surgical specimen The small, reddish-pink, well-circumscribed pedunculated soft tissue mass (right) is shown alongside the No. 15 scalpel blade used for surgical excision of the growth.

Immediate postoperative area follow-up after surgical excision of the gingival growth revealed a raw area of blood at the marginal gingiva in relation to the right maxillary lateral incisor (Figure 4).

**Figure 4:**
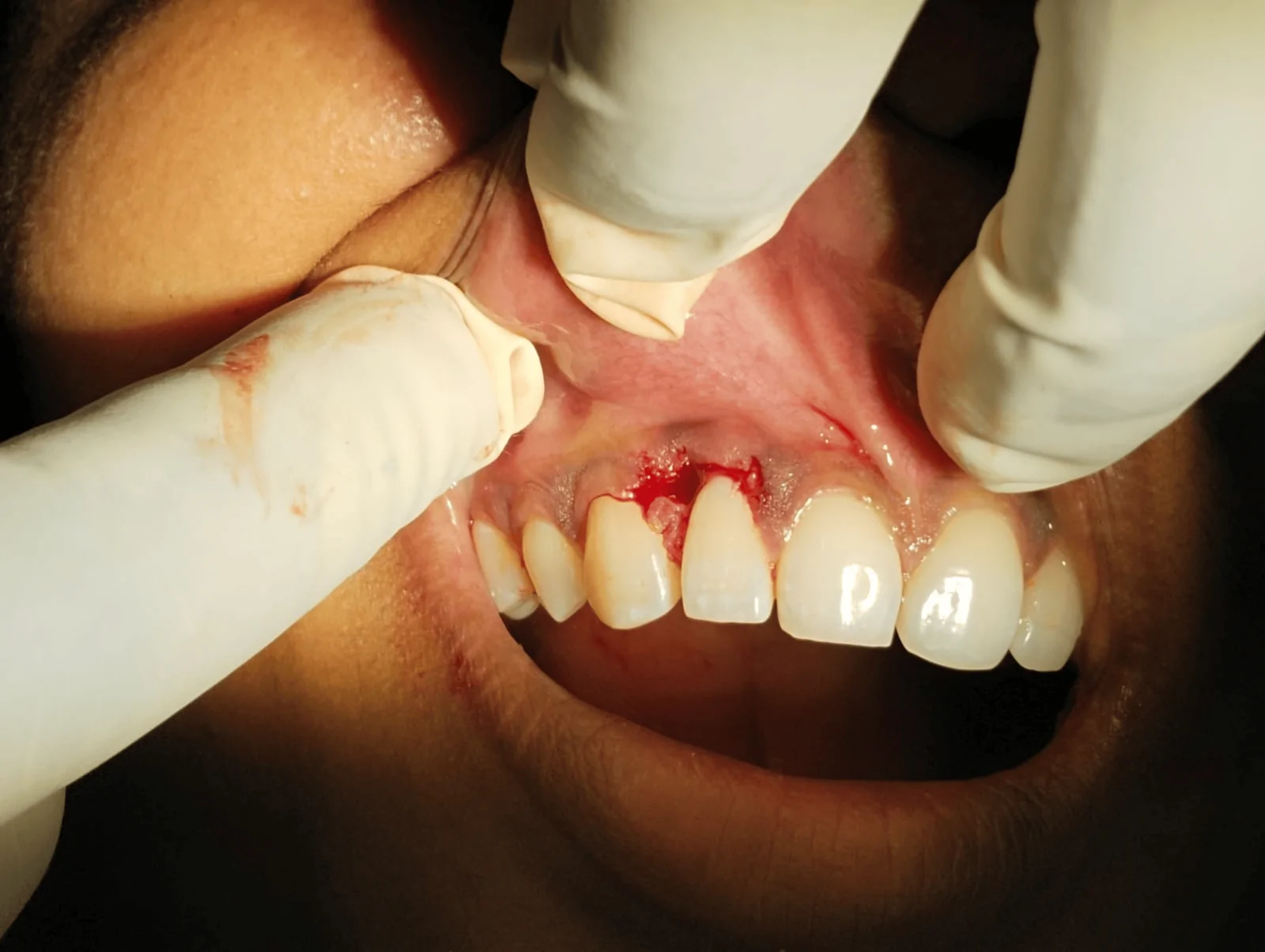
Immediate postoperative clinical photograph following surgical excision of gingival growth caused by first trimester of pregnancy leaving raw area of blood at excised surgical site of the gingival growth.

The excised surgical specimen was sent for histopathological examination. The histopathological photomicrograph (100x) revealed a well-circumscribed proliferation of granulation tissue and extravasated red blood cells (Figure 5).

**Figure 5:**
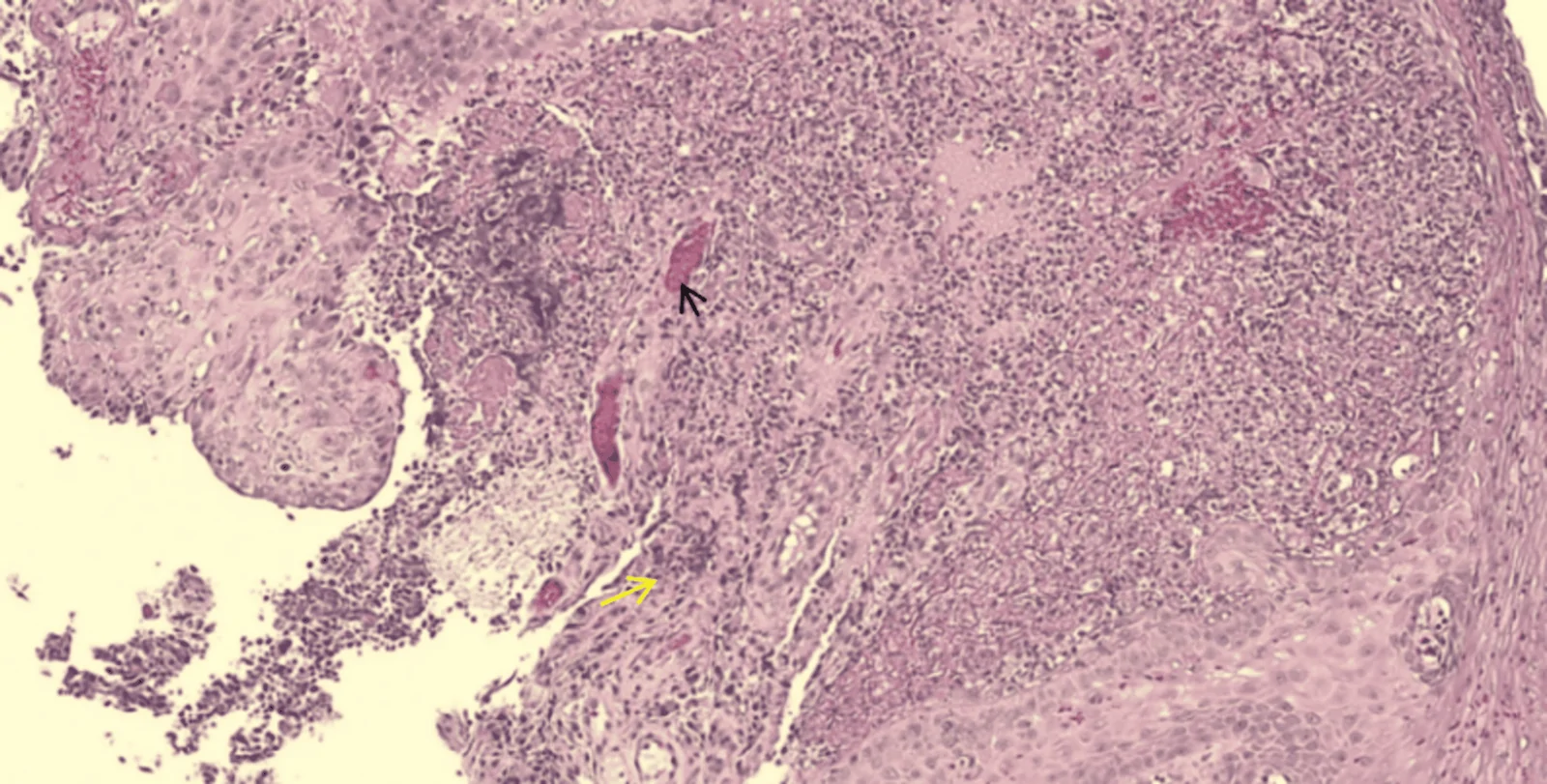
Histopathological photomicrograph (100X) revealed multiple endothelial cells in focal granulation tissue (yellow arrow) interspersed with extravasated red blood cells (black arrow)

The patient was prescribed capsule cephalexin 400 mg thrice daily for five days and tablet paracetamol 500 mg once daily for 3 days. Follow-up after a week revealed satisfactory postoperative healing, with no recurrence of the growth.

## Discussion

The pathogenesis of pregnancy-associated gingival pyogenic granuloma is considered a hormonally amplified angiogenic response to local inflammatory stimuli. Elevated estrogen levels during pregnancy enhance the responsiveness of gingival endothelial cells, macrophages, and stromal cells, increasing the production of proangiogenic mediators - particularly vascular endothelial growth factor (VEGF) - which drives endothelial proliferation, migration, and neovascularisation and accounts for the lesion’s highly vascular, hemorrhagic clinical appearance [3]. Basic fibroblast growth factor (bFGF) may further contribute to angiogenesis, while a relative reduction in antiangiogenic activity favours excessive vascular proliferation [3]. Female steroid hormones may additionally reduce apoptosis of granuloma-associated cells, prolonging the angiogenic response; following parturition, declining hormonal stimulation and VEGF expression promote endothelial apoptosis and vascular regression, which explains the spontaneous involution observed in some pregnancy tumours [3]. Thus, pregnancy tumour represents a localised interaction among plaque-induced inflammation, hormonal modulation, enhanced angiogenesis, and altered cellular survival, rather than a true neoplastic process [4,5].

While conservative management remains the gold standard during pregnancy - allowing the lesion to regress spontaneously postpartum as systemic hormone levels normalise - interventions are sometimes indicated before delivery [6,7]. This report describes the clinical diagnosis, disease course, and management of a pregnancy tumour in a 21-year-old female in the first weeks of gestation, emphasising the critical balance between maternal-fetal safety, symptom control, and structured oral care [8,9].

Serum estradiol was measured at 692 pg/mL. Reassessed against gestational-age-appropriate reference intervals rather than standard non-pregnant reference ranges (150-300 pg/mL), this value falls within the physiological range expected during the first trimester of pregnancy (approximately 188-2,497 pg/mL, varying by exact gestational week and assay), and should therefore be interpreted as consistent with normal pregnancy-associated hormonal change rather than as a pathologically elevated finding.

Follow-up after one week revealed satisfactory healing at the surgical site, with no clinical evidence of recurrence at this interval. However, this follow-up period is insufficient to draw conclusions regarding definitive treatment success, given that pregnancy-associated pyogenic granuloma is known to carry a risk of persistence or recurrence for as long as elevated hormonal stimulation continues. Longer-term follow-up extending through the remainder of pregnancy and into the postpartum period was not obtained in this case and represents a limitation of this report; the authors recommend that future reports of surgically managed pregnancy pyogenic granuloma incorporate structured follow-up extending at least through parturition to more meaningfully assess recurrence risk.

## Conclusions

Pregnancy tumours (granuloma gravidarum) are benign, highly vascular oral lesions driven by an interplay of gestational hormonal surges and local chronic irritation. While their rapid growth and tendency for profuse bleeding can cause significant patient anxiety, management should focus on maternal-fetal safety. Conservative non-surgical therapy - centred on meticulous oral hygiene instruction and local plaque control - remains the gold-standard initial approach during pregnancy, as many lesions demonstrate spontaneous regression postpartum. Surgical excision during gestation is generally reserved for cases complicated by uncontrollable haemorrhage, severe functional interference with mastication, diagnostic ambiguity, or significant patient distress and aesthetic concern that meaningfully affects the patient's quality of life, as in the present case, where a painless, non-bleeding lesion was excised primarily on the basis of aesthetic concern and patient discomfort rather than haemorrhagic or functional complications. A collaboration between obstetricians and dental professionals is imperative for providing definitive care and reducing adverse gestational outcomes.

## References

[1] Alshuhail O, Alharbi AS, Alakeel N (2023). A persistent oral pyogenic granuloma: a case report with review of literature. Cureus.

[2] Meshram M, Durge K, Shirbhate U (2023). An overview of oral pyogenic granuloma and its management: a case report. Cureus.

[3] Alarcón-Sánchez MA, González-Cárdenas JP, Martínez-González EJ, González-Alvarez GE, Victory-Rodríguez G, Heboyan A, Lomelí-Martínez SM (2026). Clinical management and surgical intervention for oral pyogenic granuloma during pregnancy: a case report. J Surg Case Rep.

[4] Alcaraz-Baturoni FJ, Ibarra-González JP, Alarcón-Sánchez MA (2026). Multifactorial etiology of oral pyogenic granuloma: case reports with distinct triggers. J Surg Case Rep.

[5] Rockafellow A, Florin W, Philipone E, Koslovsky D (2015). Pregnancy tumor in a 31-year-old female with a facial port-wine stain. Case Rep Dent.

[6] Enezei HH, Aldelaimi AA, Humadi TY (2026). The pyogenic granuloma: localization, incidence, clinical features. Minerva Dent Oral Sci.

[7] Sonar PR, Panchbhai AS (2024). Pyogenic granuloma in the mandibular anterior gingiva: a case study. Cureus.

[8] Chappuis L, Passemard L, Barthelemy I, Chevenet C, Devoize L, Pham Dang N (2023). An aggressive pregnancy epulis with extensive alveolar bone resorption and tooth displacement. J Obstet Gynaecol Res.

[9] Bi J, Sun Y, Bi L, Larjava HS (2019). Large pregnancy-associated pyogenic granuloma: a case report. J Obstet Gynaecol.

